# Effects of tumour necrosis factor-α on BrdU incorporation in cultured human enterocytes

**DOI:** 10.1155/S0962935195000068

**Published:** 1995-01

**Authors:** J. McDevitt, C. Feighery, C. O'Farrelly, G. Martin, D. G. Weir, D. Kelleher

**Affiliations:** 1 Departments of Immunology and Clinical Medicine Trinity College Dublin and St James's Hospital Dublin 8 Ireland

## Abstract

Bromodeoxyuridine incorporation is a useful method for studying the pattern of DNA synthesis in proliferating cells. The distribution pattern of incorporated BrdU in villus enterocytes of duodenal explants was analysed after exposure to TNFα in organ culture. TNFα caused a consistent, low level uptake of BrdU in the portion of the nucleus close to the nuclear membrane, this pattern was absent from the control cultures. As these epithelial cells are terminally arrested in G_0_, the BrdU incorporation was thought not to be due to S phase DNA synthesis, but rather a response to the cytotoxic influence of TNFα. Microtitre plate proliferation assays of cell density and DNA synthesis were devised to study the effects of TNFα on confluent monolayers of the human foetal jejunal cell line I407 and the mouse fibrosarcoma cell line L929. Both cell lines showed a similar response to TNFα. Exposure to TNFα alone did not reduce cell numbers but did cause a significant increase in DNA synthesis (p < 0.05). When cycloheximtde was added in tandem with TNFα there was a significant reduction in cell number (p < 0.001) and level of DNA synthesis (p < 0.01) indicative of cell death. The DNA of cells exposed to TNFα and cycloheximide was fragmented when viewed on an electrophoresis gel. The results show that BrdU incorporation might be a good indicator of damage to the DNA of cells after cytotoxic insult. TNFα may be responsible for villus enterocyte damage in enteropathies such as coeliac disease and GVHR of the small bowel.


Mediators of Inflammation 4, 31–37 (1995)

### Effects of tumour necrosis factor-α on BrdU incorporation in cultured human enterocytes

J. McDevitt, C. Feighery, C, O'Farrelly, G. Martin, D. G. Weir and D. Kelleher[CA]

Departments of Immunology and Clinical Medicine, Trinity College, Dublin and St James's Hospital, Dublin 8, Ireland

[CA] Corresponding Author

**Bromodeoxyuridine incorporation is a useful method for studying the pattern of DNA synthesis in proliferating cells. The distribution pattern of incorporated BrdU in villus enterocytes of duodenal explants was analysed after exposure to TNFα in organ culture. TNFα caused a consistent, low level uptake of BrdU in the portion of the nucleus close to the nuclear membrane, this pattern was absent from the control cultures. As these epithelial cells are terminally arrested in $G_0$, the BrdU incorporation was thought not to be due to S phase DNA synthesis, but rather a response to the cytotoxic influence of TNFα. Microtitre plate proliferation assays of cell density and DNA synthesis were devised to study the effects of TNFα on confluent monolayers of the human foetal jejunal cell line I407 and the mouse fibrosarcoma cell line L929. Both cell lines showed a similar response to TNFα. Exposure to TNFα alone did not reduce cell numbers but did cause a significant increase in DNA synthesis ($p < 0.05$). When cycloheximide was added in tandem with TNFα there was a significant reduction in cell number ($p < 0.001$) and level of DNA synthesis ($p < 0.01$) indicative of cell death. The DNA of cells exposed to TNFα and cycloheximide was fragmented when viewed on an electrophoresis gel. The results show that BrdU incorporation might be a good indicator of damage to the DNA of cells after cytotoxic insult. TNFα may be responsible for villus enterocyte damage in enteropathies such as coeliac disease and GVHR of the small bowel.**

**Key words**: Bromodeoxyuridine, Enterocyte, S phase, Tumour necrosis factor

### Introduction

The human small bowel intestinal mucosa is lined by a rapidly proliferating epithelial monolayer. The proliferative phase starts from a ring of stem cells in the lower third of the crypt and extends up to the middle third of the crypt.[1] The enterocytes overlying the villi are terminally differentiated in $G_0$ of the cell cycle and are thus incapable of dividing.[2] Increased crypt enterocyte proliferation and villus atrophy was induced in foetal jejunal explants cultured *in vitro* in the presence of pokeweed mitogen. The morphological changes in the cultured tissue were thought to have been caused by T-cell derived lymphokines.[3] In murine graft *vs.* host disease in the small intestine the extent of crypt hyperplasia and villus atrophy correlated directly with the degree of lymphocyte infiltration in the lamina propria and the villus atrophy was thought to be possibly attributable to lymphokine(s).[4]

Bromodeoxyuridine (BrdU) incorporation into DNA has been utilized to estimate the S phase labelling indices *in vivo*[5] and *in vitro*[6,7] in human colorectal mucosa. Bromodeoxyuridine has also been used in an intestinal epithelial cell line microtitre plate proliferation assay[8] and also to detect low level proliferation of peripheral blood mononuclear cells in response to the T-cell mitogen tuberculin.[9] The various subdivisions of S phase have been elucidated by immunohistochemical localization of bromodeoxyuridine in cultured tumour cell lines.[10]

The cytokine tumour necrosis factor-α (TNFα) is produced by monocytes and T cells and has multiple synergistic and pleiotrophic effects. It is thought to exert cytotoxic and proliferative effects on different cells *in vivo* and *in vitro*.[11] Induction of several nuclear events were observed, e.g. decrease of DNA synthesis, DNA fragmentation, inhibition of cell cycle progression and gene expression.[12,13] TNFα causes an increase in the number of late S phase nuclei incorporating bromodeoxyuridine in the TNF sensitive mouse fibrosarcoma cell line L929.[14]

The aim of this study was to demonstrate how exposure of organ cultured villus enterocytes to TNFα results in low level uptake of BrdU which seems to be distinct from that seen in crypt enterocytes passing through S phase of the cell cycle.

© 1995 Rapid Communications of Oxford Ltd

A microtitre plate proliferation assay using the foetal jejeunal cell line I407 was devised to quantify the cytotoxic effects of TNFα.

### Materials and Methods

*Organ culture of duodenal biopsies:* Duodenal biopsies were obtained from individuals attending the clinic for diagnostic endoscopy in whom small bowel disease was excluded. The biopsies were obtained per-endoscopically from the second stage of the duodenum. The culture medium consisted of RPMI 1640 supplemented with 10% foetal calf serum, HEPES buffer (1 M), 1 M L-glutamine, 50 μg/ml gentamicin and non-essential amino acids, 1 mM sodium pyruvate and 50 μM 2-mercaptoethanol and 2 IU/ml insulin. The medium was also supplemented with 3 ng/ml TNFα (British Biotechnology). Some biopsies were also cultured in medium without TNFα as a control. The dose of TNFα was chosen following preliminary studies which showed that TNFα induced proliferative responses in non-confluent I407 epithelial cells. The explants were cultured with TNFα for 18 h at 37°C on fine stainless steel mesh in 2 ml Falcon organ culture dishes (Becton Dickinson, Cowley, UK) in a sealed organ culture chamber (Flow Labs, Uxbridge, UK) previously infused with 95% oxygen and 5% $CO_2$. At 18 h the explant growth medium was aspirated and replaced with complete medium supplemented with BrdU (50 μM) and fluordeoxyuridine (FdU, 5 μM)[28,29] and the explants were cultured for a further 2.5 h. They were then fixed in Carnoys fixative, dehydrated in increasing concentrations of xylene in ethanol and embedded in paraffin wax. Serial sections of 3 μm were cut and dewaxed. The sections were emersed in 1 N HCl for 8 min at 60°C to allow denaturation of the DNA. The sections were then incubated in anti-BrdU antibody (1/20 Dako, Glostrup, Denmark). The binding of the antibody was visualized by a streptavidin peroxidase technique using the substrate diamino-benzidine (DAB). The slides were counterstained with haematoxylin and eosin. A coverslip was applied to the slide after adding a solvent based mounting medium. The sections were viewed by light microscopy.

*Cell lines:* I407 is an intestinal epithelial cell line initiated in 1955 from human embryonic jejunum (ATCC, Rockville, MD, USA). L929 is a mouse fibrosarcoma cell line (ATCC). The L929 line used was known to have retained its sensitivity to TNF. The cells were seeded at $10^6$/flask (Nunc, 50 ml) and passaged weekly in supplemented RPMI-1640 medium.

*MTT proliferation assay:* This was adapted from a published method.[15] L929 and I407 cells were seeded at $2 \times 10^5$/ml in flat-bottomed 96-well microtitre plates. The cells were allowed to grow to confluence over 8 days. TNFα was added at a final concentration of 3 ng/ml for a further incubation of 18 h, 100 μl of normal medium was added to control wells. The effect of the TNF on cell density was assessed using the colorimetric absorbance of solubilized MTT [3-(4,5-dimethylthiazol-2-yl)-2,5-diphenyltetrazolium bromide] crystals from the cell monolayers. Briefly, 20 μl of MTT in culture medium (6 mg/ml) was added to each well 4 h before the end of the incubation with TNF. The medium was then aspirated. The plate was dried in an oven at 50°C for 3 h. Aliquots of 150 μl of isopropyl alcohol containing 5% formic acid were added to each well to dissolve the MTT crystals. Absorbance was read on a microtitre plate reader (Titertek) at 590 nm. The assay was repeated using medium supplemented with the peptide translation inhibitor, cycloheximide (0.2 μg/ml). The assay gives an estimate of cell density.

*Bromodeoxyuridine cell line proliferation assay:* This was adapted from an ELISA technique developed for the measurement of leukocyte proliferation.[9] L929 and I407 cells were seeded at $2 \times 10^5$/ml in flat-bottomed 96-well microtitre plates. As above, the cells were allowed to grow to confluence (8 days). TFNα at a final concentration of 3 ng/ml was added to the wells with control well receiving a similar volume of culture medium. The cells were incubated for a further 18 h and then BrdU and FdU were added to each well in complete medium at a final concentration of 50 and 5 μM respectively, and cultured for a further 2.5 h. The experiment was repeated adding cycloheximide to the culture media as described above. The cell monolayers were washed several times in PBS and dried at 50°C in an oven. The cells were then fixed in absolute ethanol for 10 min. The plates were air dried overnight. 2 N HCl was added to each well to denature the cellular DNA. Then the acid was removed. 0.1 M $Na_2B_4O_7$ (pH 8.4) was added to each well to neutralize any remaining acid. The plates were then washed four times in PBS/0.5% Tween 20 (PBST). Anti-BrdU was added to each well (1/75, Dako, Glostrup, Denmark) and incubated at room temperature for 1 h. PBST with mouse serum was added as a negative control for the ELISA. The plates were then washed several times with PBST. Peroxidase conjugated rabbit anti-mouse IgG (Dako) was added to each well at a concentration of 1/1000 in PBST. Orthophenylenediamine (OPD) in citrate buffer (0.003% $H_2O_2$) was used as substrate for the ELISA. The reaction was allowed to proceed in the dark for 10 min and was then stopped by adding 50 μl of 2 N HCl to each well. Absorbance was read at 450 nm using a Titertek multiscan plate reader. The BrdU plate assay was taken to indicate the levels of DNA synthesis proceeding in the cell monolayers.

A preliminary validation exercise was carried out for the two assays to see how they compared with each other. Microtitre plates were seeded with I407 cells for 9 consecutive days with four different concentrations of foetal calf serum. The plates were processed in duplicate to compare cell density with DNA synthesis over the 9 day period (Fig. 2). Results for the MTT and BrdU validation assays were expressed as mean ± S.D. of 12 microtitre wells (Fig. 2). Results of the MTT and BrdU TNF assays in Fig. 3 were expressed as mean ± S.E.M. (assays were repeated three to eight times on different days). Each assay consisted of the mean ± S.D. of three to six microtitre well absorbance readings.

*DNA extraction and electrophoresis:* The DNA of I407 and L929 cells exposed to TNF (with or without cycloheximide) was extracted to assess any cytotoxic effects of the TNF by adapting a published technique.[16] Briefly, the cells were pelleted at 350 ×***g*** for 10 min and taken up in 1.5 ml of $H_2O$ and 200 μl of SDS. This solution was allowed to lyse the cells for 10 min before 1.5 ml of resuspension buffer (20 mM Tris, 800 mM NaCl and 4 mM EDTA, pH 8.2) was added. Lysis buffer (250 μl, 2% SDS, 4 mM EDTA) was added and then the cellular protein were digested overnight at 37°C with 250 μl protease K (Sigma, 5 mg/ml). The proteins were precipitated by gently shaking in 1 ml of saturated NaCl (6 M) for 15 s and centrifuging at 1 600 ×***g*** for 15 min. The supernatant containing the DNA was decanted into a fresh tube and 2.5 volumes of 100% ethanol was added. The DNA was spooled out with a wire loop and dissolved in T:E buffer (10:1, Tris, 800 mM; EDTA, 80 mM; pH 8.0). The DNA concentration was estimated from the optical density at 260 nm of a 1:200 dilution. Samples containing 4 μg of DNA were made up to 15 μl in double distilled $H_2O$, and an equal volume of denaturing tracking dye was added (50% formamide, 20% bromophenol blue (1%) stock, 30% TAE (0.04 M Tris acetate, 0.01 M EDTA, pH 7.9). The DNA was heated to 70°C for 1 h to denature the DNA duplex, rapidly ice cooled, and electrophoresed through 1% agarose gel. Undenatured and denatured *Hin*dIII digested Lambda DNA (BRL) were used as fragment size markers. The acridine orange and ethidium bromide stained gels were illuminated using UV and photographed.

### Results

*Villus enterocyte BrdU incorporation:* Figure 1a shows granular, immunoperoxidase staining of the nucleus of the surface enterocytes of a duodenal explant after 18 h culture in medium containing TNFα. This is indicative of BrdU uptake into newly synthesized DNA. The staining is located close to the nuclear membrane ('perinuclear'). This staining is faint in comparison to that seen in the crypt enterocytes engaged in S phase of mitosis (Fig. 1c). The staining is absent from the enterocytes from the control cultures (i.e. no TNF in the culture medium (Fig. 1b)). Some of the underlying lamina propria lymphocytes also show relatively strong BrdU uptake (Fig. 1a). The faint granular perinuclear stain in the surface enterocytes approximates closely in appearance to the late S phase configurations (S4 and S5) described by Van Dierendonck *et al.* in cultured

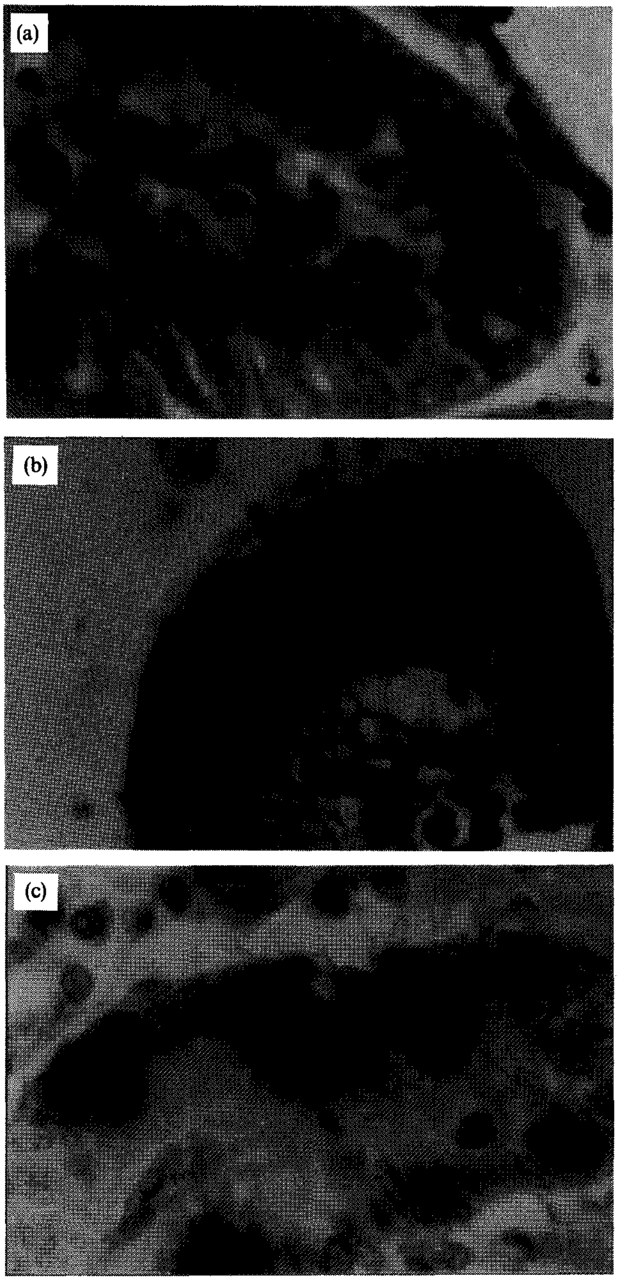

FIG. 1. (a) Villus enterocytes showing low level uptake of BrdU after exposure to TNFα in culture (×1 900). The replication points are largely confined to the nuclear membrane. The BrdU uptake is most obvious in enterocytes at the extreme tip of the villus. (b) Villus tip enterocytes from the control culture showing negligible BrdU uptake (×1 900). (c) Crypt enterocytes showing high level BrdU uptake during S phase after a similar culture period in normal medium. All sections were visualized with the same immunoperoxidase/DAB staining protocol.

tumour cell lines[10] and to the Type 3 pattern of BrdU incorporation observed in L929 cells exposed to TNF.[14] These findings were consistently observed in seven control individuals.

*Time course of DNA synthesis and cell population growth in I407 cells cultured to confluence:* Peak DNA synthesis occurs on day 5 with medium containing 2% and 5% FCS and on day 4 with medium

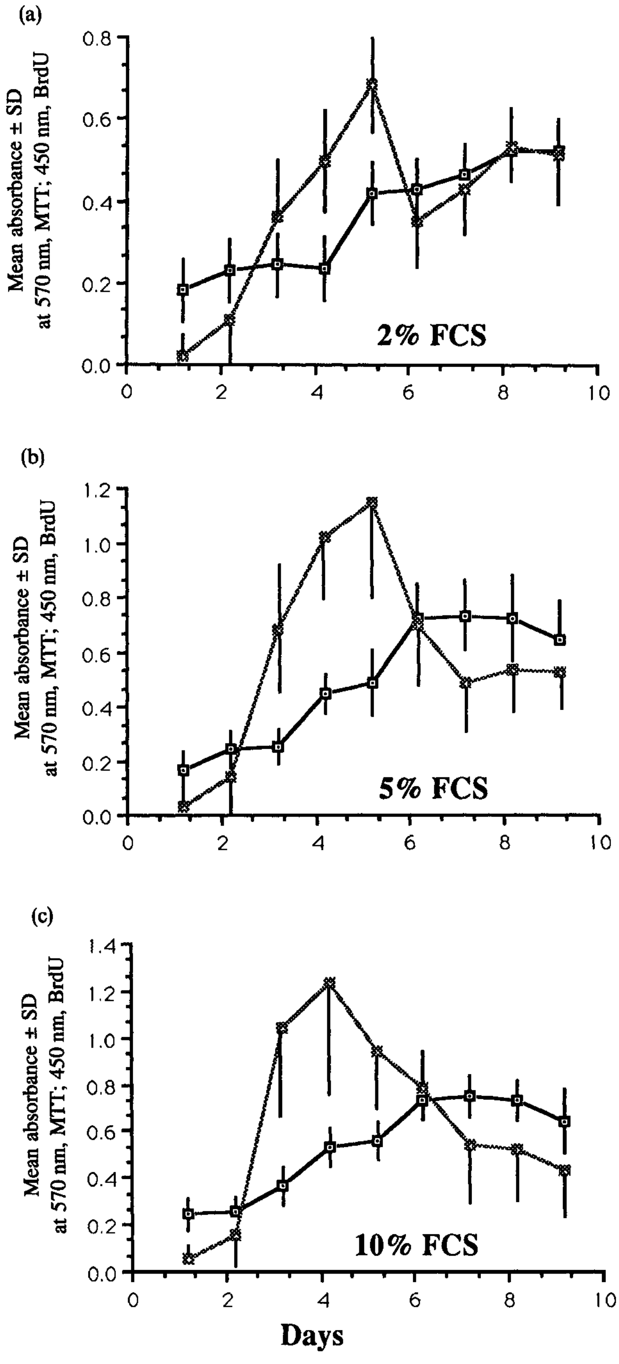

FIG. 2. A comparison of the MTT cell density and BrdU DNA synthesis assays using I407 epithelial cell line over time and different concentrations of foetal calf serum in the culture medium. The assays were carried out in parallel at the same time. The cells reach maximum density (confluence) towards days 7 and 8. However, peak DNA synthesis occurs during the log phase of growth on days 4–5 when most of the cells are in S phase of the cell cycle. [·], MTT assay; [ ], BrdU assay.

containing 10% FCS (Fig. 2). Maximum DNA synthesis occurs in medium containing 10% FCS. Peak DNA synthesis probably occurs when most of the cell population is engaged in S phase during the 'log phase' of population growth. In contrast, maximum cell density does not occur until days 7 and 8, by which time DNA synthesis has subsided to a background level. At this stage most of the cells have left S phase and have reached a quiescent state of $G_1$ or $G_0$ in the cell cycle.

*TNFα induced DNA synthesis in confluent I407 and L929 cells:* The MTT assay, which relates to the number of viable cells, showed no significant difference in cell density of the I407 cells cultured in TNF relative to those cultured in normal medium; x ± S.E.M., 0.597 ± 0.112 *vs.* 0.582 ± 0.123, $n = 7$, $p$, NS. However, when the media was supplemented with an inhibitor of protein synthesis, cycloheximide, there was a significant decrease in the cell density of I407 cells exposed to TNF relative to the controls: x ± S.E.M., 0.188 ± 0.058 *vs.* 0.335 ± 0.119, $n = 3$, $p < 0.001$ (Fig. 3a).

The BrdU incorporation assay shows a significant increase in DNA synthesis in the I407 cells exposed to TNF relative to those cultured in control medium. x ± S.E.M., 0.655 ± 0.214 *vs.* 0.529 ± 0.210, $n = 8$, $p < 0.05$. However, when the media was supplemented with cycloheximide there was a decrease in DNA synthesis in the cells exposed to TNF relative to those cultured in control medium: x ± S.E.M., 0.175 ± 0.083 *vs.* 0.804 ± 0.093, $n = 3$, $p < 0.01$ (Fig. 3b).

The MTT assay showed a slight decrease in cell density in L929 cell exposed to TNF relative to those cultured in normal medium; x ± S.E.M., 0.401 ± 0.045 *vs.* 0.534 ± 0.046, $n = 3$, $p$, NS. However, when the media was supplemented with cycloheximide there was a significant decrease in the cell density of those cells exposed to TNF relative to those cultured in the control medium: x ± S.E.M., 0.106 ± 0.034 *vs.* 0.329 ± 0.042, $n = 3$, $p < 0.001$ (Fig. 3c).

The BrdU assays showed an increase in the level of DNA synthesis in those cells exposed to TNF relative to those cultured in the control medium: x ± S.E.M., 0.795 ± 0.177 *vs.* 0.732 ± 0.218, $n = 3$, $p < 0.05$ (Fig. 3d). In summary, this data shows that the response of the two cell lines to TNFα is essentially the same. Exposure to TNF alone did not reduce cell density (except for a slight decrease for L929 cells), however it did cause a significant increase in DNA synthesis. When cycloheximide is added in tandem with TNF there was a significant reduction in cell number and the level of DNA synthesis in both cell lines.

*DNA fragmentation in I407 and L929 cells on exposure to TNFα:* After 16 h exposure to TNFα the DNA of the cell lines was extracted and denatured such that single-stranded nicks in the damaged DNA du-

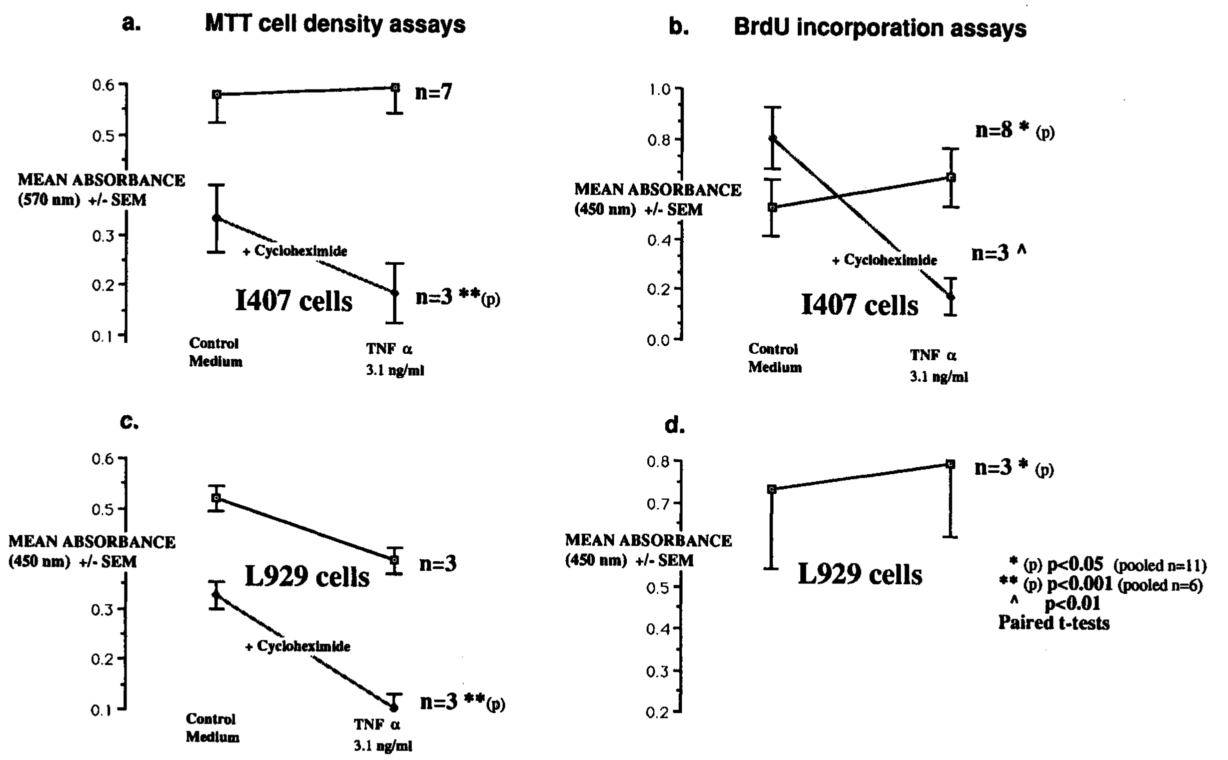

FIG. 3. All cell cultures were confluent. The response of the I407 and L929 cells to TNFα is essentially the same, (MTT assays a and c). When the cell lines are cultured with TNF there is almost no change in cell density (apart from the L929 cells which do show a slight decrease in density, Fig. 1c). However, when cycloheximide is added to the media, there is a marked decrease in cell density in the cells exposed to TNF, indicative of cell death ($p < 0.001$). (BrdU assays b and d). There is a significant increase in DNA synthesis in those cells cultured in TNF ($p < 0.05$). When the cultures were repeated with media supplemented with cycloheximide there was marked reduction in DNA synthesis in the I407 cells ($p < 0.01$). The paired $t$-test was used to compare the two culturing regimens; medium +TNFα and control medium.

plex could be visualized by agarose gel electrophoresis. The DNA of the cells exposed to TNFα was largely intact when compared with the controls. However, when the protein translation inhibitor cycloheximide was added to the culture medium the DNA of the I407 and L929 cell lines showed a significant degree of DNA fragmentation as evidenced by the presence of low molecular weight DNA close to the cathode end of the electrophoresis gel (Fig. 4).

### Discussion

On exposure to TNFα the villus enterocytes of the cultured duodenal explants exhibited conspicuous low level uptake of BrdU suggestive of newly synthesized DNA. It was noted that the replication granules were localized for the most part close to the nuclear membrane. However, there were a few villus enterocytes at the extreme villus tip also showing larger irregularly shaped replication granules throughout the whole nucleus. These observations are in keeping with previous observations for late S phase in cultured cell lines.[10,14,17] The villus enterocytes are terminally differentiated cells arrested in $G_0$ of the cell cycle, therefore the DNA synthesis cannot be attributable to S phase. It is plausible to suggest that this DNA synthesis could be due to the

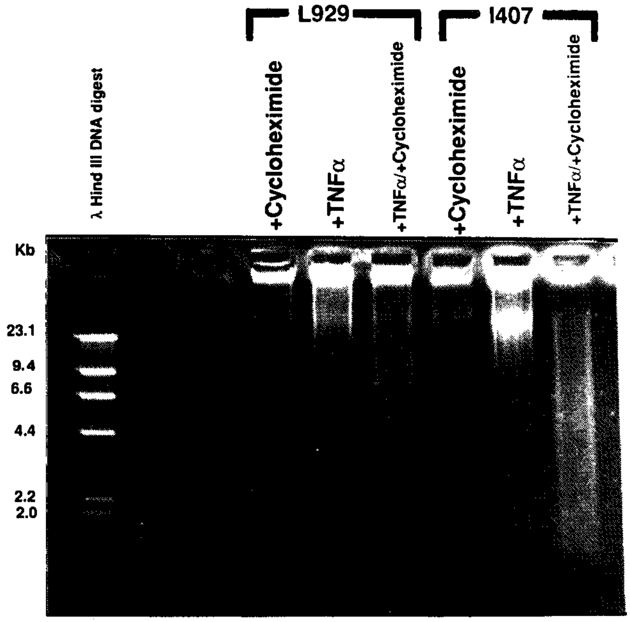

FIG. 4. Agarose gel showing fragmented DNA of I407 and L929 cells after 16 h exposure to TNF and/or cycloheximide. Undamaged high molecular weight DNA remains close to, or in, the anode well. Fragmented DNA of a variety of smaller molecular weights are apparent as smears down the electrophoresis gel.

same process of unscheduled DNA synthesis as that previously described in tumour cell lines cultured in the presence of TNF.[8] In this case the authors proposed that the unscheduled DNA synthesis was attributable to DNA repair after the cytotoxic effects of TNF.

Similar unscheduled DNA synthesis has been reported in a number of experimental conditions. DNA repair occurs in bovine lens epithelial cells after exposure to $H_2O_2$. The repair process involving new DNA synthesis is completed within 30 min of the noxious insult.[9] Similarly, DNA synthesis due to repair occurs over a period of 50 min in $H_2O_2$ exposed or UV irradiated human epithelioid P3 cells.[20] TNF in association with elicited macrophages was shown to induce sub-lethal DNA damage in cultured mammary tumour cells and this damage was potentiated in the presence of actinomycin D. This damage could be partially abrogated by the addition of inhibitors of arachidonate metabolism, removal of $H_2O_2$ with catalase, or addition of antibodies to TNFα.[18] Cycloheximide augmented DNA damage and apoptosis in TNFα exposed U937 tumour cells relative to cells cultured in TNF alone.[21] Human recombinant TNFα was shown to induce DNA damage and attendant apoptosis in cultured bovine endothelial cells.[22] Exposure of cultured L929 cells to TNF reduced the proportion of BrdU positive cells. However, exposure to TNF in late S phase resulted in increased numbers of what the authors termed 'type 3 BrdU distribution patterns' i.e. large irregularly shaped replication granule clusters distributed throughout the whole nucleus.[14] The same effect was absent when a TNF resistant L929 variant cell line was used. A number of explanations were put forward to explain this phenomenon, i.e. changes in the speed of successive events during S phase, by influence of TNF on the nuclear matrix or on enzymes involved in DNA replication. It is known that TNF affects the activity of a $Ca^{2+}$ dependent endonuclease[3] and DNA polymerase α[24] and topoisomerase II.[25,26] All these enzymes are capable of causing strand breakages in DNA. It could be argued that the late S phase L929 cells of Kraeft *et al.*[14] might have left S phase altogether and that the type 3 BrdU distribution pattern that they observed might be analogous to the type of pattern in Fig. 1a.

To further investigate the phenomenon of unscheduled DNA synthesis in the small bowel biopsy explants on culturing with TNFα, an *in vitro* assay was developed to demonstrate the damaging effect of TNFα on the foetal jejunal stem cell line I407 grown to confluence on 96-well-microtitre plates. The mouse fibroblast tumour cell line with known sensitivity to TNF was used as a positive control for the assay. Recombinant TNFα at a concentration of 3 ng/ml or control medium was added to each of the duplicate plates. After 18 h the effect of TNFα was assayed using the MTT assay (a measure of cell density) and a BrdU incorporation ELISA technique (a measure of DNA synthesis). BrdU incorporation has been used previously to measure DNA synthesis due to repair processes after ultraviolet radiation induced damage in cultured human fibrolasts.[27] It transpired that the I407 cell line was as sensitive to the effects of TNFα as the L929 cell line. The MTT assays showed little difference in cell density in those cells cultured in TNFα relative to those cultured in normal medium. However, in parallel, the BrdU assays showed a significant increase in DNA synthesis in those cells exposed to TNFα in comparison with those cells cultured in normal medium ($p < 0.05$). It was concluded that the DNA synthesis must have been due to DNA repair processes as there was no increase in cell density (but rather a slight decrease). When the experiments were repeated with the addition of cycloheximide to the culture media, virtually all the cells exposed to TNF underwent apoptosis ($p < 0.001$, MTT assays; $p < 0.01$, BrdU assays) while those cultured in the control medium survived despite the presence of cycloheximide. Agarose gel electrophoresis revealed significant DNA fragmentation in the DNA of those cells exposed to the combination of TNFα and cycloheximide, as predicted by the microtitre plate assays. The DNA of the cells cultured separately in TNFα and cycloheximide was largely intact suggesting that the protein translation inhibitor cycloheximide blocks DNA repair in the cell lines. Thus, the otherwise sub-lethal dose of TNFα became lethal. This suggests that the ability of cell nuclear DNA to repair itself after exposure to TNFα is dependent on *de novo* peptide translation. By inference from these experiments it is possible that the subtle BrdU uptake in the villus epithelium of explants cultured in TNFα is due to ongoing DNA synthesis associated with DNA damage/repair.

In conclusion, it is possible that TNFα mediates a sub-lethal DNA injury signal to villus enterocytes with resultant DNA repair as evidenced by their BrdU uptake subsequent to TNF exposure. TNFα at a concentration of 3 ng/ml induces DNA synthesis in the intestinal epithelial cell line I407 grown to confluence. The DNA synthesis may be due to DNA repair as opposed to S phase. The cells are normally able to survive exposure to TNF. The presence of the protein translation inhibitor, cycloheximide, abrogates the ability of I407 cells to repair damaged DNA on exposure to an otherwise sub-lethal dose of TNFα, in tandem with other factors could be responsible for villous atrophy in conditions such as celiac disease and graft *vs.* host disease of the small intestine. More studies are required to more clearly define the mechanism whereby TNF influences the cellular differentiation and metabolism of the small bowel epithelium.

### References

1. Wright NA, Watson A, Morley A, Appleton D, Marks J, Douglas A. Cell kinetics in flat avillous mucosa of the human small intestine. *Gut* 1973; **14**: 701–710.
2. Wright NA, Allison M. The kinetic organisation of the gut epithelia. In: *The Biology of Epithelial Cell Populations, Volume 1*. Oxford: Clarendon Press, 1984: 688–740.

3. Ferreira M, Forsyth LE, Richman PI, Wells C, Spenser J, MacDonald TT. Changes in the rate of crypt epithelial cell proliferation and mucosal morphology induced by a T-cell mediated response in the human small intestine. *Gastroenterology* 1990; **98**: 1255–1263.
4. Mowat AM, Felstein MV. Experimental studies of immunologically mediated enteropathy during an acute graft versus host reaction in adult BDF1 mice. *Clin Exp Immunol* 1990; **79**: 279–284.
5. Potten CS, Kellet M, Roberts SA, Row DA, Wilson GD Measurement of *in vivo* proliferation in human colorectal mucosa using bromodeoxyuridine. *Gut* 1992; **3**: 71–78.
6. Risio M, Coverlissa S, Ferrari A, Candelaresi G, Rossini FP. Immunohistochemical study of epithelial cell proliferation in hyperplastic polyps, adenomas and adenocarcinomas of the large bowel. *Gastroenterology* 1988, **94**: 899–906.
7. Wilson RG, Smith AN, Bird CC. Immunohistochemical detection of abnormal cell proliferation in colonic mucosa of subjects with polyps. *J Clin Pathol* 1990; **43**: 744–747.
8. Lowes JR, Priddle JD, Jewell DP. Production of epithelial cell growth factors by lamina propria mononuclear cells. *Gut* 1992; **33**: 39–43.
9. Huong PL, Kolk AH, Eggelte TA, Verstijnen CP, Gilis H, Hendricks JT. Measurement of antigen specific lymphocyte proliferation using 5-bromodeoxyuridine incorporation. *J Immunol Methods* 1991; **140**: 243–248.
10. Van Dierendonck JH, Keyser CH, Van de Velde C, Cornelisse J. Subdivisions of S phase by analysis of nuclear 5-bromodeoxyuridine staining patterns. *Cytometry* 1989; **10**: 143–150.
11. Semenzato G. Tumour necrosis factor: a cytokine with multiple biological activities (review). *Br J Cancer* 1990; **61**: 354–361.
12. Fiers W. Tumour necrosis factor: characterisation at the molecular, cellular and *in vivo* level. *FEBS Lett* 1991; **285**: 199–212.
13. Kleine HD, Wagner H, Poliwoda H, Freund M. Effects of TNFα on bone marrow aspirates of patients with acute myelogenous leukaemia determined by flow cytometric cell cycle analysis. *J Cancer Res Clin Oncol* 1992; **118**: 56–60.
14. Kraeft SK, Rychly J, Walschlager U, Nebe B, Schutt W. Bromodeoxyuridine patterns in L929 fibroblasts and influence of tumour necrosis factor. *Eur J Cell Biol* 1993; **62**: 415–421.
15. Mosmann T. Rapid colorimetric assay for cellular growth and survival: application to proliferation and cytotoxicity assays. *J Immunological Methods* 1983; **65**: 55–63.
16. Miller SA, Dykes DD, Polesky HF. A simple salting out procedure for extracting DNA from human nucleated cells. *Nucleic Acid Res* 1988; **16**: 1215.
17. Fox MH, Arndt-Jovin D, Jovin TM, Baumann PH, Robert-Nicoud D. Spatial and temporal distribution of DNA replication sites localised by immunofluorescence and confocal microscopy in mouse fibroblasts. *J Cell Sci* 1991, **99**: 247–253.
18. Fulton AM, Chong YC. The role of macrophage derived tumour necrosis factor α (TNFα) in the induction of sublethal tumour cell DNA damage. *Carcinogenesis* 1992; **13**: 77–81.
19. Spector A, Kleiman NJ, Huang R, Wang RR. Repair of hydrogen peroxide induced DNA damage in bovine lens epithelial cell cultures. *Exp Eye Res* 1989; **49**: 685–698.
20. Peak JG, Pilas B, Dudek EJ, Peak MJ. DNA breaks caused by monochromatic 365nm UV-A radiation or hydrogen peroxide and their repair in human epithelioid and xeroderma pigmentosum cells. *Photochem Photobiol* 1991; **54**: 197–203.
21. Wright SC, Kumar P, Tam AN, Shen N, Varma M, Larrick JW Apoptosis and DNA fragmentation precedes TNF induced cytolysis in U937 cells. *J Cell Biochem* 1992; **48**: 344–355.
22. Robaye B, Moselmans R, Fiers W, Dumont JE, Galand P. Tumour necrosis factor induced apoptosis of programmed cell death in normal endothelial cells *in vitro*. *Am J Pathol* 1991; **138**: 447–453.
23. Bellomo G, Perotti M, Taddei F, Mirabelli F, Finardi G, Nicotera P, Orrenius S. Tumor necrosis factor α induces apoptosis in mammary adenocarcinoma cells by an increase in intranuclear free $Ca^{2+}$ concentration and DNA fragmentation. *Cancer Res* 1992; **52**: 1342–1346.
24. Santavenere E, Cataldi A, Rana R, *et al.* Nuclear metabolic changes induced by tumour necrosis factor in Daudi lymphoma cells: a multiparametric analysis. *Cell Biol Int Rep* 1991; **15**: 1235–1242.
25. Alexander RB, Nelson W, Coffey D. Synergistic enhancement by tumour necrosis factor of *in vitro* cytotoxicity from chemotherapeutic drugs targeted at DNA topoisomerase II. *Cancer Res* 1987; **47**: 2403–2406.
26. Utsugi T, Mattern N, Mirabelli C, Hanna N. Potentiation of topoisomerase induced DNA strand breakage and cytotoxicity by tumour necrosis factor: enhancement of topoisomerase activity as a mechanism of potentiation. *Cancer Res* 1990; **50**: 2636–2640.
27. Cohn SM, Leiberman MW. The use of antibodies to 5'-bromo-2'-deoxyuridine for the isolation of DNA sequences containing excision repair sites. *J Biol Chem* 1984; **259**: 12456–12462.
28. Dormer P, Ellwart J. Effect of 5-fluoro-2'-deoxyuridine (FdU) on 5-bromo-2'-deoxyuridine (BrdU) incorporation into DNA measured with a monoclonal BrdU antibody and the BrdU Hoecht quenching effect. *Cytometry* 1985; **6**: 513–520.
29. Meyer JS, Nauert J, Koehm S, Hughes J. Cell kinetics of human tumours by *in vitro* bromodeoxyuridine labelling. *J Histochem Cytochem* 1989; **37**: 1449–1454.

ACKNOWLEDGEMENTS. D.K. is a Wellcome Senior Fellow in Clinical Science. C.O'F. is supported by the Health Research Board.

**Received 6 September 1994; accepted 27 September 1994**
